# Combination of FIB-4 with ultrasound surface nodularity or elastography as predictors of histologic advanced liver fibrosis in chronic liver disease

**DOI:** 10.1038/s41598-021-98776-1

**Published:** 2021-09-29

**Authors:** Maryam Moini, Fernanda Onofrio, Bettina E. Hansen, Oyedele Adeyi, Korosh Khalili, Keyur Patel

**Affiliations:** 1 Division of Gastroenterology and Hepatology, University Health Network, University of Toronto, Toronto General Hospital 9EN, 200 Elizabeth Street, Toronto, ON M5G 2C4 Canada; 2 Department of Laboratory Medicine and Pathology, University of Minnesota, Minneapolis, USA; 3 Department of Joint Medical Imaging, University Health Network, University of Toronto, Toronto, Canada

**Keywords:** Gastroenterology, Medical research

## Abstract

Reliable and available non-invasive methods for hepatic fibrosis assessment are important in chronic liver disease (CLD). Our aim was to compare stepwise algorithms combining standard ultrasound with serum markers and transient elastography (TE) for detecting advanced fibrosis (F3-4) and cirrhosis. Retrospective single center study between 2012 and 2018 of CLD patients with biopsy, TE, blood tests, and liver ultrasound parameters of surface nodularity (SN), lobar redistribution, and hepatic vein nodularity. Our cohort included 157 patients (51.6% males), mean age 47.6 years, predominantly non-alcoholic fatty liver disease and viral hepatitis (61%), with F3-4 prevalence of 60.5%. Area under the curve for F3-4 was 0.89 for TE ≥ 9.6 kPa and 0.80 for FIB-4 > 3.25. In multivariate modeling, TE ≥ 9.6 kPa (OR 21.78) and SN (OR 3.81) had independent association with F3-4; SN (OR 5.89) and TE ≥ 10.2 kPa (OR 15.73) were independently associated with cirrhosis. Two stepwise approaches included FIB-4 followed by SN or TE; sensitivity and specificity of stepwise SN were 0.65 and 1.00, and 0.89 and 0.33 for TE ≥ 9.6 kPa, respectively. Ultrasound SN and TE were independently predictive of F3-4 and cirrhosis in our cohort. FIB-4 followed by SN had high specificity for F3-4.

## Introduction

Cirrhosis is the most advanced form of chronic liver disease (CLD), and the 12th most common cause of death globally^1^. The global health burden of cirrhosis is expected to increase significantly over the next two decades due to the increasing prevalence of non-alcoholic fatty liver disease (NAFLD), although end-stage liver disease due to chronic viral hepatitis is declining due to the availability of safe and effective antivirals, and preventive measures^2,3^. Assessment of advanced disease in CLD is important for both prognosis, monitoring the response to treatment, and surveillance measures for hepatocellular carcinoma and complications of portal hypertension. Liver biopsy remains the reference standard for fibrosis assessment but is invasive, associated with serious potential complications, and subject to sampling errors and interobserver variability^4–6^.

Non-invasive modalities for assessment of hepatic fibrosis are now increasingly used in clinical practice for management decisions in patients with chronic liver disease. Simple serum tests for advanced fibrosis include FIB-4^7^ or AST to platelet ratio index (APRI)^8^. Imaging-based methods include Vibration-controlled transient elastography (VCTE) as a validated method using liver stiffness measurement for diagnosis of advanced fibrosis and cirrhosis in CLD^9^. VCTE is a point-of-care test, and increasingly available for assessment of patients with CLD. Other imaging methods such as Magnetic Resonance Elastography (MRE) have high accuracy in staging advanced fibrosis, but it is expensive with limited availability^10,11^. Standard B-mode Ultrasound (US) is a widely available modality obtained as routine assessment in patients with CLD. Liver surface nodularity (SN), lobar redistribution (LRD), and hepatic vein nodularity (HVN) can be observed during examination as morphologic features suggestive of cirrhosis^12,13^. Thus, combining routine US assessment with VCTE or a simple serum marker as FIB-4 may allow for increased non-invasive diagnostic accuracy for advanced disease.

Our aim was to develop and compare stepwise algorithms combining standard ultrasound parameters with simple serum markers or transient elastography for the detection of advanced fibrosis and cirrhosis.

## Methods

This is a single centre retrospective study with convenience sampling that evaluated patients with CLD at University Health Network Toronto between January 2012 and December 2018.

### Participants

All adult CLD patients (age > 18) with liver biopsies performed at University Health Network Toronto and available liver US performed through our Department of Medical Imaging, University Health Network Toronto within study defined period were included. Exclusion criteria were: repeated liver biopsies from one single patient, inadequate or poor quality VCTE tests, acute liver injury, targeted biopsy of mass or lesion, history of metastatic cancer and post-transplant status and history of previous hepatic resection (Supplementary Figure 1).

### Histopathological assessment

Liver biopsies were reported by two expert hepatopathologists at University Health Network Toronto that were unblinded to patients’ clinical information for these standard-of-care biopsies. Specific interobserver variability for fibrosis staging was not evaluated in this study. Adequacy of sample size was defined as at least one core of liver tissue of 20 mm, or considered adequate for fibrosis staging by our tertiary center histopathologists. All biopsies were stained by the hematoxylin & eosin and trichrome stains and included only when deemed adequate for fibrosis staging. The modified Metavir (Laennec) staging system was used for reporting^14^.

### Ultrasound

US reports were reviewed for three main parameters used to define hepatic morphologic changes of cirrhosis including: SN, LRD and HVN as described previously^12,15,16^. Operators were unblinded to the patients’ clinical information at the time these standard-of-care ultrasounds were obtained.

### VCTE

VCTE (EchoSens, Paris, France) was performed in fasting state by experienced operators using M or XL probes. Requirement for a valid test were at least 10 liver stiffness measurements using the appropriate probe and IQR/Med ≤ 30%. Operators were unblinded to the patients’ clinical information, but at our center VCTE is typically performed by a technician without an interest in patient level information. Only valid measurements within 6 months of liver biopsy were considered for the study. For patients having multiple US exams or VCTE within the defined interval, the closest interval to biopsy was considered for analysis.

### Serum markers

Blood work results available within 6 months of liver biopsies were used for calculation of APRI and FIB-4 scores. APRI was calculated using our statistical software according to Wai et al.^8^ and validation by Lin et al.^17^ FIB-4 was calculated using our statistical software according to Sterling et al.^7,8,17^.

This study was performed according to the protocol and guidelines of Good Clinical Practice/ICH, based upon the principles outlined in the Declaration of Helsinki and local and national guidelines governing the conduct of clinical research studies. All patient data used for the purpose of this retrospective study was de-identified and anonymized, and patient consent was not required. This study was approved by the University of Toronto Research Ethics Boards (REBs). Waiver for inform consent was obtained from REBs.

Data were reported in accordance with the Standards for Reporting Diagnostic accuracy studies (STARD) 2015 statement.

### Statistical analysis

Statistical analysis was carried out using statistical package for social sciences (SPSS) (IBM Corp. Released 2017. IBM SPSS Statistics for Windows, Version 25.0. Armonk, NY: IBM Corp.). Student’s t-test was used to compare the means of variables between each two groups. Pearson’s Chi-square test and univariate regression were used for assessment of association of categorical variables with dependent variables. Continuous variables are shown as means (± standard deviation). Backward elimination logistic regression was used to assess for independent variables for the prediction of advanced fibrosis and cirrhosis. Area Under Receiver operating characteristic (AUROC) curve was used to demonstrate the predictive performance of non-invasive continuous variables for advanced fibrosis and cirrhosis. We included validated thresholds for APRI > 2 for cirrhosis, and FIB-4 > 3.25 for prediction of advanced fibrosis. Statistical significance was assessed at *P* < 0.05 level.

## Results

### Patient demographics

A total of 178 CLD patients with available liver biopsies, VCTE, and US within 6 months of liver biopsy were evaluated. Of these, 21 cases were excluded for: targeted lesion biopsy, non-diagnostic, inadequate or no liver tissue, diagnosis of lymphoma, sarcoidosis, graft-versus-host disease or acute hepatitis, and 157 cases were included in the final analysis (supplementary Figure 1).

The study population comprised of marginally increased proportion of males (81/157; 51.6%) and mean age of 47.6 ± 14.7 years (Table 1). The main etiology of liver disease was NAFLD (33.8%) followed by chronic viral hepatitis (26.8%) comprising predominantly Hepatitis B (38/42; 90%) and Hepatitis C (4/42; 10%)). Most patients with chronic hepatitis B (35/38; 92%) and all four chronic hepatitis C patients were treatment naïve at the time of biopsy. Based on histology, 95/157 (60.5%) patients in our cohort had advanced fibrosis (stage F3-4), and 67/157 (42.7%) had cirrhosis (stage F4).

**Table 1:** Demographic and clinical characteristics of the study cohort.

Demographic	Number of patients (%)
Male sex (%)	81 (51.6%)
Age, mean (± SD) years	47.61 (± 14.7)
**Etiology of liver disease**	
NAFLD	53 (33.8%)
Viral Hepatitis	42 (26.8%)
Cholestatic liver disease	15 (9.6%)
Autoimmune Hepatitis	9 (5.7%)
Other diagnoses (including NYD)	32 (20.4%)
Normal liver biopsies	6 (3.8%)
**Liver fibrosis stage histology**	
F0	27 (17.2%)
F1	20 (12.7%)
F2	15 (9.6%)
F3	28 (17.8%)
F4	67 (42.7%)
**VCTE**	
Mean LSM (± SD) kPa	15.47 (± 14.51)
≥ 9.6 kPa	82 (52.2%)
> 10.2 kPa	75 (47.8%)
**Ultrasound parameters**	
Liver surface nodularity	46 (29.3%)
Lobar redistribution	38 (24.5%)
Hepatic vein nodularity	28 (18.1%)

kPa, kilo pascal; LSM, liver stiffness measurement; NAFLD, non-alcoholic fatty liver disease; NYD, not yet determined; SD, standard deviation; VCTE, vibration controlled transient elastography.

### VCTE

Mean liver stiffness measurement (LSM) for patients with cirrhosis in our cohort was 24.95 ± 17.53 kPa and 20.93 ± 16.26 kPa for advanced fibrosis. As expected, LSM showed a significant association (95% CI 0.04–0.07; *P* < 0.001) with fibrosis stage F0-4.

The AUROC for VCTE and advanced fibrosis was 0.89 (95% CI 0.84–0.94; *P* < 0.001) and for cirrhosis 0.89 (95% CI 0.84–0.94; *P* < 0.001) (Supplementary Figure 2 A and B). The optimal cut-off levels of LSM for advanced fibrosis and cirrhosis were selected as 9.6 kPa and 10.2 kPa, respectively (Table 2).

**Table 2:** Diagnostic performance of VCTE for advanced fibrosis and cirrhosis.

	AF(F3-F4) (LSM ≥ 9.6 kPa)	Cirrhosis (F4) (LSM ≥ 10.2 kPa)
Sensitivity	78.9%	85.1%
Specificity	88.7%	80.0%
PPV	91.5%	76.0%
NPV	73.3%	87.8%
AUROC (95% CI)	0.89 (0.84–0.94)	0.89 (0.84–0.94)

AF, advanced fibrosis; AUROC, area under receiver operating characteristic curve; CI, confidence interval; LSM, liver stiffness measurement; NPV, negative predictive value; PPV, positive predictive value; VCTE, vibration controlled transient elastography.

### Ultrasonography

US cirrhosis features of HVN, LRD and SN were seen in 35.9%, 47.7% and 53% of patients with cirrhosis, respectively; 45/67 (67.2%) of cirrhotic patients had at least one of these three US features. In univariate analyses, all three US parameters assessed in this study showed significant association with histology confirmed F3-4 and F4 (*P* < 0.001 for both). However, US parameters had low sensitivity (34–55%) and negative predictive values (NPV) for the diagnosis of cirrhosis, with higher corresponding specificity (> 90%) and positive predictive (PPV) values (Table 3). In a multivariate logistic regression including all three US parameters, SN (OR 5.67; 95% CI 2.24–14.32; *P* < 0.001) and LRD (OR 4.58; 95% CI 1.65–12.70; *P* = 0.003) showed significant independent associations with cirrhosis. However, only SN (OR 5.29; 95% CI 1.78–15.75; *P* = 0.003) but not LRD showed a significant independent association with advanced fibrosis (F3-F4). HVN was not independently associated with F3-4 or F4 in our multivariate logistic regression model.

**Table 3:** Diagnostic performance of ultrasound parameters for cirrhosis (F4).

	Hepatic vein nodularity (%)	Lobar redistribution (%)	Nodular surface (%)
Sensitivity	33.8	55.2	47.0
Specificity	93.3	90.0	92.1
PPV	78.6	80.4	81.6
NPV	66.1	73	70

NPV, negative predictive value; PPV, positive predictive value.

### Combined US and VCTE model prediction for advanced fibrosis

We performed multivariate analyses to investigate the interaction between US parameters and VCTE for prediction of advanced fibrosis. In a multivariate logistic regression for the prediction of advanced fibrosis including HVN, LRD, SN and VCTE at LSM ≥ 9.6 kPa, only VCTE with LSM ≥ 9.6 kPa and SN showed independent significant association with advanced fibrosis with ORs of 21.78 and 3.81, respectively (Table 4). Based on our multivariate model, the combination of both US SN and LSM ≥ 9.6 kPa resulted in a predicted value of 96.40% for advanced fibrosis. This predicted value is 87.19% when LSM was ≥ 9.6 kPa but no SN is reported on US, 54.56% when there is US SN but LSM < 9.6 kPa, and 23.34% when SN is negative and LSM < 9.6 kPa.

**Table 4:** Multivariable regression model to assess ultrasound parameters and VCTE for advanced fibrosis and cirrhosis.

Variables	Advanced fibrosis (F3-4)			Cirrhosis (F4)		
	OR	95% CI	*P* value	OR	95% CI	*P* value
HVN	2.82	0.38–20.86	0.310	0.309	0.05–1.95	0.211
LRD	0.72	0.16–3.20	0.670	2.507	0.77–8.17	0.127
SN	3.81	1.16–12.50	0.027	5.886	2.16–16.015	0.001
VCTE^a^	21.78	8.44–56.22	< 0.001	15.732	6.46–38.30	< 0.001

HVN, hepatic vein nodularity; LRD, lobar redistribution; OR, odd’s ratio; SN, surface nodularity; VCTE, vibration controlled transient elastography.

^a^Liver stiffness measurement ≥ 9.6 kPa for F3-4 and ≥ 10.2 kPa for F4.

We evaluated the diagnostic performance of combining US parameters of SN and VCTE with LSM ≥ 9.6 kPa for advanced fibrosis. This combined approach resulted higher specificity and PPV compared to either test alone, but at the expense of decreased sensitivity and NPV, with 17% of patients misclassified (Table 5).

**Table 5:** Diagnostic performance of combined ultrasound surface nodularity and VCTE for advanced fibrosis and cirrhosis.

Combined US-SN and VCTE^a^	Sensitivity (%)	Specificity (%)	PPV (%)	NPV (%)	+ LR	− LR	Misclassified (%)
F3-4	38.9	98.4	97.4	51.3	6.99	0.24	17.2
F4	50.7	96.7	91.8	72.5	4.25	0.19	17.8

LR, likelihood ratio; NPV, negative predictive value; PPV, positive predictive value; SN, surface nodularity; VCTE, vibration controlled transient elastography.

^a^Liver stiffness measurement ≥ 9.6 kPa for F3-4 and ≥ 10.2 kPa for F4.

### Combined US and VCTE model prediction for cirrhosis

Our multivariate model for Cirrhosis indicated SN and LSM ≥ 10.2 kPa were independently associated with F4 with OR of 5.89 (95% CI 2.16–16.02) and 15.73 (95% CI 6.46–38.30), respectively (Table 4).

The combination of both SN and LSM ≥ 10.2 in the multivariate regression model resulted in a predicted value of 90.79% for F4. The predictive value for F4 was reduced with the absence of SN and LSM ≥ 10.2 kPa (61.60%), SN present and LSM < 10.2 kPa (37.88%). The absence of SN and LSM was associated with predictive value of 9.03% for cirrhosis.

Similar to the model for advanced fibrosis, combining SN and VCTE ≥ 10.2 kPa for cirrhosis, resulted in increase in specificity and PPV compared to either test alone, but decreased sensitivity and a comparable proportion of misclassified patients (Table 5).

### Diagnostic performance of simple serum markers

We next evaluated the diagnostic utility of APRI and FIB-4 scores, which were available in 140 CLD patients with prevalence of 61.4% and 42.1% for F3-4 and F4 respectively. Separate analyses were performed for each of these two tests for association with advanced fibrosis and cirrhosis.

In univariate analysis, APRI test as a continuous variable did not show a significant association with either advanced fibrosis (*P* = 0.888) or cirrhosis (*P* = 0.860). However, as a categorical variable APRI > 2 showed a significant association with both advanced fibrosis (*P* = 0.034) and cirrhosis (*P* = 0.042). The mean APRI score for F0-2 (1.39 ± 4.17) was not significantly different from the mean score for F3-4 fibrosis (1.47 ± 2.59), (*P* = 0.497). The same was true for mean APRI scores for F0-3 compared to F4 (1.48 ± 4.14 vs. 1.38 ± 1.41; *P* = 0.147). Using established APRI thresholds and after excluding 28 patients in the indeterminate zone for F4 (APRI score 1–2), the AUROC for cirrhosis was a modest 0.67 (95% CI 0.56–0.77; *P* = 0.004). For cirrhosis, APRI > 2 showed sensitivity and specificity of 25% and 93% respectively, with PPV of 66.7% and NPV of 68.8% (supplementary Figure 3).

FIB-4 as a continuous variable showed a significant association with both advanced fibrosis (*P* < 0.001) and cirrhosis (*P* < 0.001) in univariate analysis. FIB-4 > 3.25 was significantly associated with both advanced fibrosis (*P* = 0.001) and cirrhosis (*P* < 0.001). The mean FIB-4 score for F3-4 was higher as compared to F0-2 (3.10 ± 3.22 vs. 1.34 ± 1.28; *P* < 0.001). The same was also true of FIB-4 scores for cirrhosis compared to non-cirrhotic (F0-3) patients (3.65 ± 3.57 vs 1.52 ± 1.47; *P* < 0.001). After excluding 45 cases (32%) in the indeterminate zone of FIB-4 index score 1.45–3.25, the sensitivity and specificity of FIB-4 > 3.25 for the diagnosis of advanced fibrosis was 47.3% and 92.5%, and PPV of 89.7% and NPV of 56.1%, respectively. The AUROC for FIB-4 and advanced fibrosis was 0.80 (95% CI 0.71–0.89; *P* < 0.001)) (supplementary Figure 4).

### Stepwise diagnostic algorithms for advanced fibrosis

**Figure 1:**
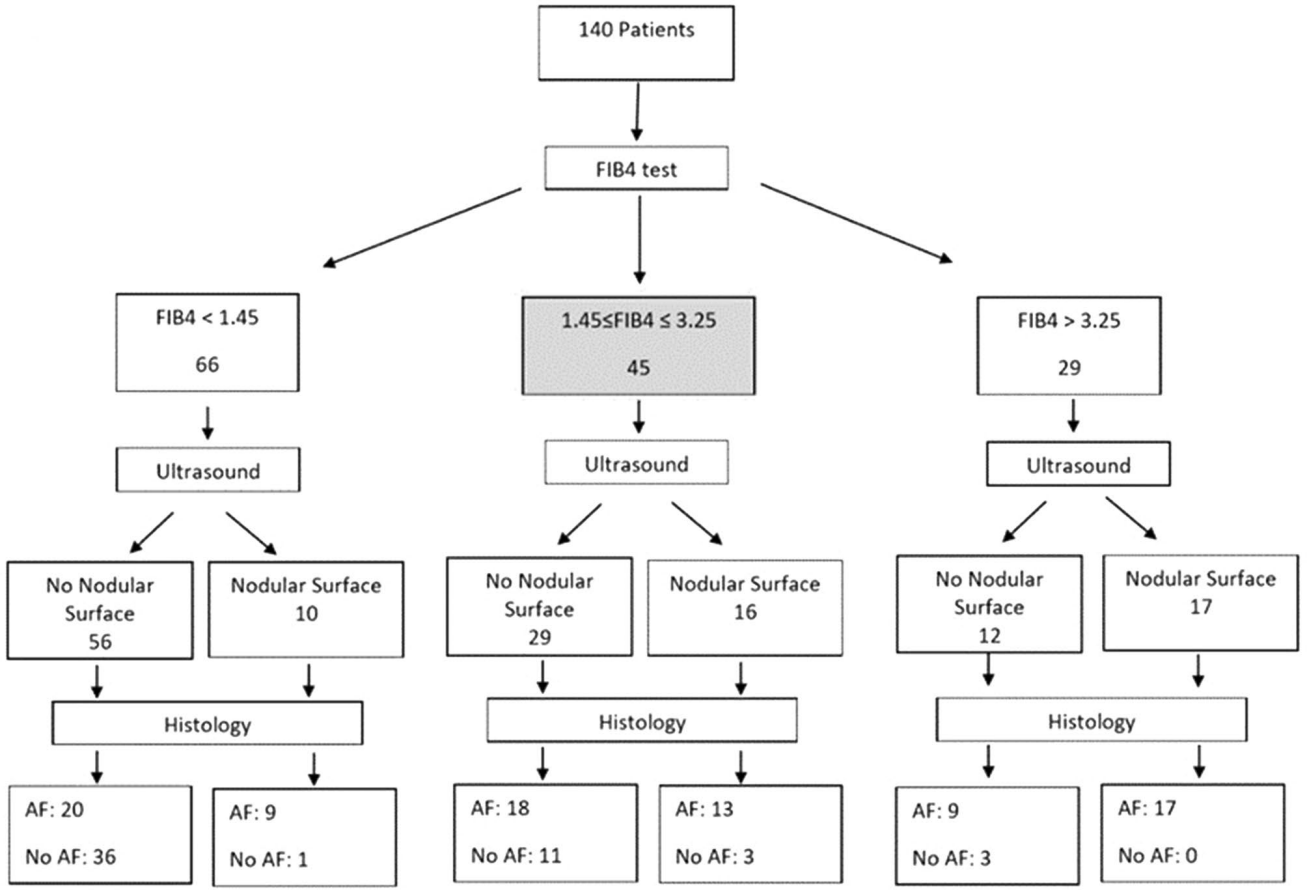
Algorithm 1: Non-invasive approach using FIB-4 and US for assessment of advanced fibrosis in patients with chronic liver disease.

**Figure 2:**
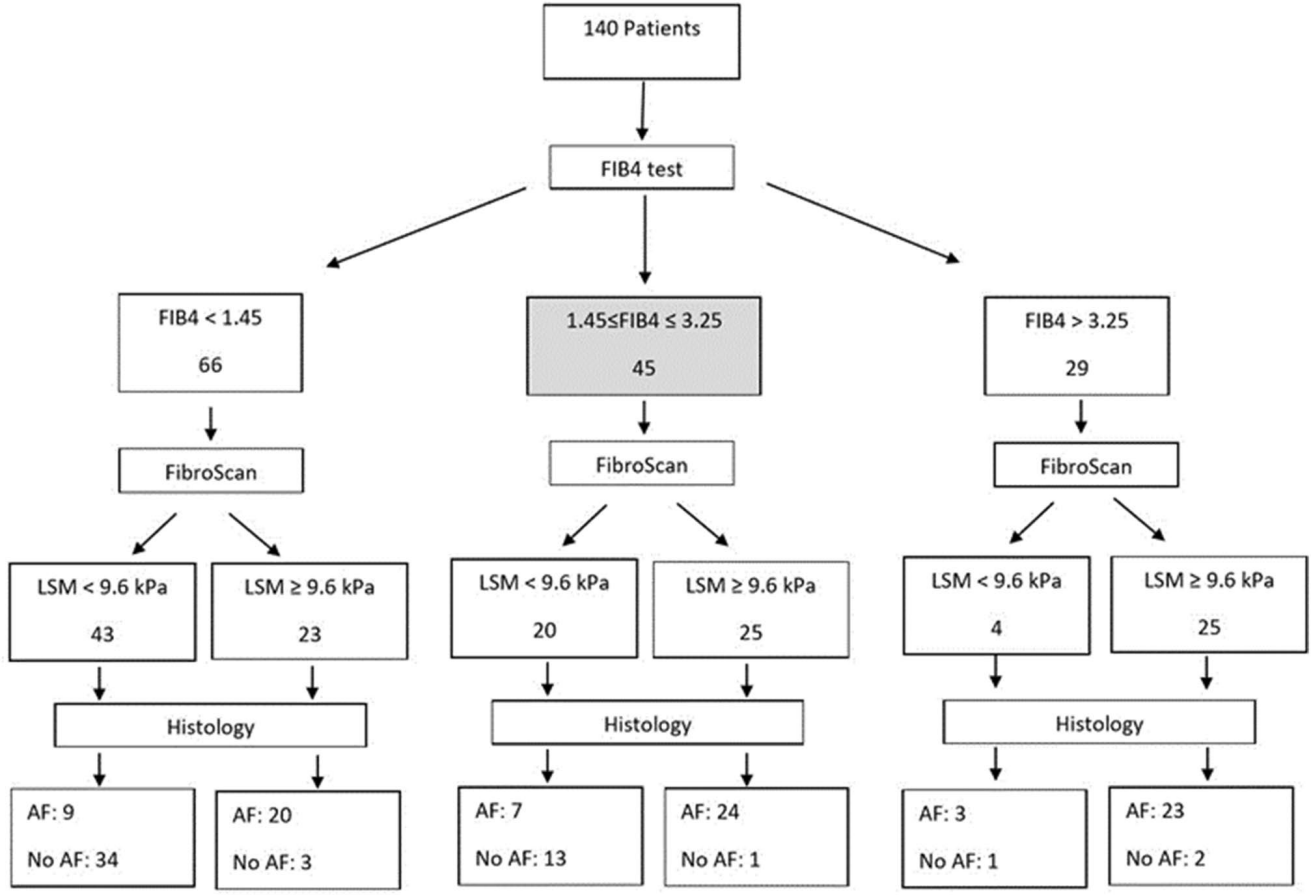
Algorithm 2: Non-invasive approach using FIB-4 and VCTE for assessment of advanced fibrosis in patients with chronic liver disease.

To optimize the prediction for advanced fibrosis and cirrhosis in our cohort, we evaluated stepwise algorithmic approaches combining imaging variables (SN or LSM) with FIB-4 for assessment of advanced fibrosis and cirrhosis, summarized as flowcharts in Figs. 1 and 2. The first non-invasive test we used for assessment of fibrosis in all 140 patients was FIB-4, chosen as an easily accessible simple test with high sensitivity at < 1.45 for ruling-out advanced fibrosis, and high specificity at > 3.25 for ruling-in advanced fibrosis, followed by a second imaging parameter (SN or LSM). Algorithm 1 included SN (Fig. 1) and algorithm 2 included LSM (Fig. 2) as the second-line tests. Based on FIB-4 scores, 66 (47.1%) patients were classified as non-advanced fibrosis (FIB-4 < 1.45), 29 (20.7%) as advanced fibrosis (> 3.25) and 45 (32.1%) in the indeterminate score range of 1.45–3.25.

In algorithm 1, patients with FIB-4 scores > 3.25, the sensitivity and specificity of US parameter of SN for prediction of advanced fibrosis were 65.4% and 100% respectively with AUROC 0.83. For patients with FIB-4 scores < 1.45, the sensitivity and specificity of SN for advanced fibrosis were 31% and 97.3% respectively, and lower AUROC 0.64. For the one-third of patients with indeterminate FIB-4 scores, the sensitivity and specificity for F3-4 fibrosis were 41.9% and 78.6%, respectively, but lower accuracy with AUROC 0.60 (Table 6). Thus, the addition of SN to FIB-4 indeterminate scores allowed for correct identification of advanced fibrosis in 13/16 (81%) of patients in the presence of SN, and exclusion of 11/29 (38%) without advanced fibrosis in the absence of SN. However, 21/45 (46%) would have been misclassified, predominantly as being false positive for advanced fibrosis in 18/21(86%) of these patients with indeterminate FIB-4 scores.

**Table 6:** Performance of stepwise algorithms of FIB-4 followed by ultrasound surface nodularity or VCTE for prediction of advanced fibrosis.

First non-invasive test	FIB-4 < 1.45		FIB-4: 1.45–3.25		FIB-4 > 3.25	
Second non-invasive test	Ultrasound (SN)	VCTE	Ultrasound (SN)	VCTE	Ultrasound (SN)	VCTE
Sensitivity	31%	69%	41.9%	77.4%	65.4%	88.5%
Specificity	97.3%	91.9%	78.6%	92.9%	100%	33.3%
PPV	90.0%	87.0%	81.3%	96.0%	100%	92.3%
NPV	64.2%	79.0%	37.8%	64.9%	24.3%	24.3%
AUROC (95% CI)	0.64 (0.51–0.76)	0.80 (0.69–0.89)	0.60 (0.45–0.74)	0.85 (0.71–0.94)	0.83 (0.64–0.94)	0.61 (0.41–0.78)

AUROC, area under receiver operating characteristic curve; CI, confidence interval; kPa, kilo pascal; LSM, liver stiffness measurement; NPV, negative predictive value; PPV, positive predictive value; SN, surface nodularity; VCTE, vibration controlled transient elastography.

In algorithm 2, VCTE with LSM ≥ 9.6 kPa indicated a sensitivity and specificity of 88.5% and 33.3% with AUROC 0.61 for advanced fibrosis in patients with FIB-4 scores > 3.25, 69% and 91.9% with AUC 0.80 in those with FIB-4 scores < 1.45, and 77.4% and 92.9% with AUROC 0.85 for the 45 patients with indeterminate range FIB-4 scores of 1.45–3.25 (Table 6). Compared to SN alone, VCTE as a stepwise test at the selected LSM resulted in higher sensitivity and maintained a specificity of > 90% for FIB-4 < 1.45. For patients with FIB-4 > 3.25, stepwise LSM ≥ 9.6 kPa had greater sensitivity of 88%, but very low specificity of 33% compared to stepwise SN (Table 6). For the 45 patients with indeterminate FIB-4 scores, LSM ≥ 9.6 kPa allowed for correct identification of advanced fibrosis in 24/31 (77.41%) and correct exclusion of 13/14 (92.86%) without advanced fibrosis in patients with LSM < 9.6 kPa. The misclassification rate for LSM following FIB-4 in patients with indeterminate scores was 17.78% and compares to 46.67% for SN.

## Discussion

In our single center study with biopsy-proven CLD, we show that simple US parameters such as SN are associated with advanced fibrosis and cirrhosis, and in combination with simple markers such as FIB-4 or imaging elastography, US provides improved diagnostic utility that may reduce the need for liver biopsy. This study is unique in proposing a combination of easily available routine tests such as simple biomarkers and US, for a stepwise approach to advanced fibrosis assessment in CLD with variable etiology.

Standard B-mode US is a simple, inexpensive and widely available imaging modality that is usually included in the initial assessment of patients with elevated liver enzymes and CLD^18,19^. However, the sensitivity of US for diagnosis of advanced fibrosis/cirrhosis is modest and its accuracy is debated^20–22^. There are multiple US parameters defined as the features of advanced hepatic fibrosis. Surface nodularity has been reported as the most sensitive feature of significant fibrosis by Choong et al.^22^ In our study, three US parameters of HVN, LRD and SN showed poor sensitivity but good specificity > 0.90 for detection of cirrhosis. In our multivariate analysis, SN showed an independent association with advanced fibrosis. In a prior study, US features of cirrhosis, including SN, showed modest predictive utility for diagnosis of cirrhosis with PPV of 68%^23^. In a systematic review on the accuracy of US for detection of CLD by Allan et al. liver surface assessment showed a moderate diagnostic accuracy to identify CLD^21^. Another study indicated that quantitative measurement of SN on CT scan could accurately differentiate cirrhotic and non-cirrhotic patients with chronic hepatitis C^24^.

Our study population compromised of a high prevalence of advanced fibrosis (60.5%), and variable CLD etiology, but mostly included NAFLD and viral hepatitis as expected for CLD patients with biopsy in tertiary clinical practice. Although liver biopsies have an important role in clinical practice for the identification of advanced NASH, due to our limited cohort, we were not able to perform further subgroup analyses on NAFLD patients. Despite our variable CLD etiology, based on a high prevalence of advanced fibrosis in our cohort, a LSM of 9.6 kPa provided the optimal threshold for prediction of advanced fibrosis with AUROC of 0.89 for advanced fibrosis. This is comparable to a summary AUROC of 0.88 observed with variable thresholds in a prior meta-analysis of 22 NAFLD studies^25^. Interestingly, a LSM threshold of 9.5 kPa has been recommended for ruling-out advanced fibrosis in chronic hepatitis C that have achieved SVR^26^, but in general, there are variable LSM thresholds for other causes of CLD as chronic hepatitis B or AIH included in our cohort^26^. Our optimal LSM of 10.2 kPa for prediction of cirrhosis in all-cause CLD are also comparable with Baveno VI recommendations that transient elastography < 10 kPa in the absence of other known clinical signs rules out advanced CLD^27^.

In our cohort, combining two independent variables of US reported SN and LSM of > 9.6 kPa had a 96.4% predictive value for advanced fibrosis. In a prior study by Zhang et al. used a specific US scoring system including scores for liver contour, liver parenchyma echotexture, hepatic vein contour and spleen size, for assessment of fibrosis in chronic hepatitis B patients with NAFLD, combining US and transient elastography. They showed the combination of the two methods significantly increased the PPV for detection of advanced fibrosis or cirrhosis compared to transient elastography alone; however, the accuracy of diagnosis was not significantly increased^28^. In our cohort combined SN and VCTE resulted in higher specificity and PPV compared to either test alone.

In our region, VCTE is principally available at specialist centers and usually associated with non-reimbursed costs. Both APRI and FIB-4 test are simple, cheap, and easy to calculate scores from routinely obtained serum tests in CLD patients. Both tests were developed and validated for non-invasive fibrosis assessment in patients with viral hepatitis and HIV-HCV co-infection^7–9,29^. FIB-4 has now been validated for first line screening to exclude advanced fibrosis in NAFLD patients^30^. These simple marker tests are not as well validated in other forms of CLD^9^. For chronic hepatitis B, FIB-4 overall diagnostic value for fibrosis is not high and is affected by the cut-off threshold^31^. Similarly, the accuracy of APRI for prediction of fibrosis in chronic hepatitis B may not be acceptable^32^. Another important drawback of these simple serum markers includes indeterminate scores in 30–40% of patients, further limiting their use as single tests in routine clinical practice^11,33^. In our cohort of mixed CLD patients with high prevalence of advanced fibrosis, with main etiology of NAFLD and viral hepatitis, FIB-4 showed a higher accuracy AUROC for prediction of advanced fibrosis compared to APRI, and was selected as the first line simple marker test for stepwise testing.

The combination of more than one non-invasive modality may increase diagnostic accuracy for detection of significant or advanced fibrosis, and using different unrelated non-invasive tests may be preferable^34,35^. Our stepwise algorithms combined FIB-4 as a simple serum marker in combination with the US parameter of SN or VCTE for assessment of advanced fibrosis. Our novel algorithm 1, using US surface nodularity as the second test, showed high specificity and PPV for detection of advanced fibrosis across all FIB-4 score, and importantly including the FIB-4 scores in the grey zone of 1.45–3.25. However, as expected, based on our results for US parameters for cirrhosis, sensitivity of SN also remained poor for advanced fibrosis. However, all patients with advanced fibrosis by FIB-4 and SN were correctly identified with no false positive results, suggesting potential utility for SN as a second line test for ruling-in advanced fibrosis in CLD without the need for biopsy. In algorithm 2 which VCTE was used as the second non-invasive test, the specificities and PPVs for advanced fibrosis were good for FIB-4 scores < 1.45 and values in the grey zone. PPV was also good for FIB-4 score > 3.25 but specificity was low, due to a very small sample, with only one of three patients without advanced disease being correctly identified by the second-line VCTE. However, VCTE showed higher sensitivity for advanced fibrosis in all ranges of FIB-4 compared with US surface nodularity, suggesting greater utility for VCTE as second-line test to rule-out advanced fibrosis. Stepwise approaches for non-invasive assessment of hepatic fibrosis were originally proposed and validated in HCV patients^34–36^. Paggi et al. proposed a 94% diagnostic accuracy for the combination of APRI test and US SN in HCV for the presence or absence of advanced fibrosis or cirrhosis in their study, but did not evaluate a stepwise approach or assess for advanced fibrosis^36^. Other stepwise algorithmic approaches have been proposed in patients with CLD such as the eLIFT-FM^VCTE^ algorithm using simple parameters followed by more complex second-line tests with prognostic implications^37^. More recently stepwise algorithms combining simple and complex serum tests or imaging elastography have been proposed to improve accuracy for advanced fibrosis in NAFLD^38,39^. These stepwise diagnostic approaches also appear to have clinical utility in selecting at-risk patients in community cohorts for referral to specialist care in NAFLD^40^. However, standard B-mode US is more routinely available than complex second-line markers such as ELF has not been previously evaluated in stepwise algorithms for NAFLD. Although we did not assess diagnostic performance in our NAFLD subgroup, the overall high specificity of US SN in our cohort suggests a potential role of US as a secondary test to rule-in advanced fibrosis for patients with high FIB-4 without need for biopsy, or for settings where VCTE is not readily available. Following FIB-4, VCTE had higher sensitivity than US SN in our cohort, and this is not unexpected based on association of quantitative LSM across the spectrum of fibrosis severity.

Limitations of our study include that this was a retrospective study at a tertiary center with for-cause biopsy and a higher prevalence of F3-4 fibrosis. Our limited cohort size did not allow for assessing important patient variables such as advanced age, body habitus, or ethnicity which may reduce diagnostic performance of non-invasive tests. Heterogeneity of CLD etiology could be another limitation of our study. Interobserver concordance and reliability of reporting US parameters such as surface nodularity across centers is not established, and external validation of these findings is important. Both M and XL probes were used for VCTE in this study to reflect clinical practice in CLD patients, and measurements were obtained by experienced operators. Another limitation is the timeframe of up to 6-months between biopsy and non-invasive tests in our study that may reduce accuracy of non-invasive tests due to changes in liver inflammatory injury based on natural history or therapeutic intervention. However, most patients with CHB and all hepatitis C patients in our cohort were treatment naive prior to biopsy, and NAFLD constituted one-third of all patients. We did not review changes in management in our other CLD patients that could have impacted simple biomarker tests and VCTE.

In summary, the proposed stepwise application of FIB-4 followed by US to assess SN showed good specificity for diagnosis of advanced fibrosis in a population of CLD patients with high prevalence of advanced fibrosis. Non-patented biomarker tests and liver ultrasound are easily available, relatively inexpensive, and simple to perform tests that are not restricted to Hepatology clinics at tertiary centers. Further validation of diagnostic approaches using US parameters in community cohorts with lower prevalence NAFLD advanced fibrosis, and chronic viral hepatitis patients following antiviral therapy, is still required.

The data that support the findings of this study are available on request from the corresponding author. The data are not publicly available due to privacy or ethical restrictions.

## Supplementary Information

Supplementary Information 1.

Supplementary Information 2.

Supplementary Information 3.

Supplementary Information 4.

Supplementary Information 5.
