# Supplementary material for: Combination of FIB-4 with ultrasound surface nodularity or elastography as predictors of histologic advanced liver fibrosis in chronic liver disease
Source: Sci Rep. 2021 Sep 29;11:19275. doi: 10.1038/s41598-021-98776-1 (PMC8481285; doi:10.1038/s41598-021-98776-1)
Supplement: Supplementary file 1 — Supplementary Information 1. [file 41598_2021_98776_MOESM1_ESM.docx]

Supplementary Figures

Supplementary Fig 1. Flow chart of cases reviewed, included based on inclusion criteria and excluded based on exclusion criteria to the final step of including 157 cases.

Supplementary Fig 2. Receiver Operating Characteristic curve (ROC) derived for VCTE with advanced fibrosis (A) and cirrhosis (B) in 157 patients with chronic liver disease.

Supplementary Fig 3. Receiver Operating Characteristic curve (ROC) derived for APRI score with cirrhosis in patients with chronic liver disease, excluding patients in the gray one score range of 1-2 (total number: 112).

Supplementary Fig 4. Receiver Operating Characteristic curve (ROC) derived for FIB-4 score with advanced fibrosis in 140 patients with chronic liver disease, excluding patients in the gray one score range of 1.45-3.25 (total number: 95).
