# Supplementary figures and images for: Combination of FIB-4 with ultrasound surface nodularity or elastography as predictors of histologic advanced liver fibrosis in chronic liver disease
Source: Sci Rep. 2021 Sep 29;11:19275. doi: 10.1038/s41598-021-98776-1 (PMC8481285; doi:10.1038/s41598-021-98776-1)

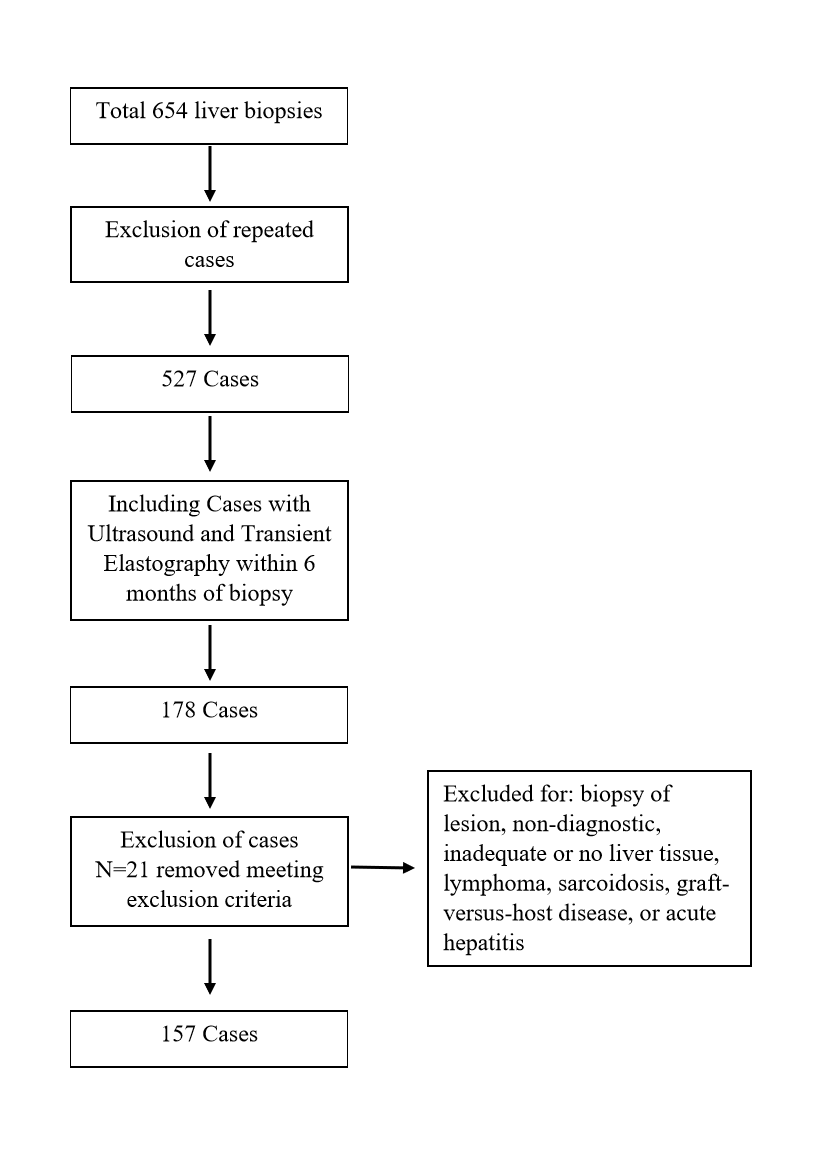

Supplement: Supplementary file 2 — Supplementary Information 2. [file 41598_2021_98776_MOESM2_ESM.png]

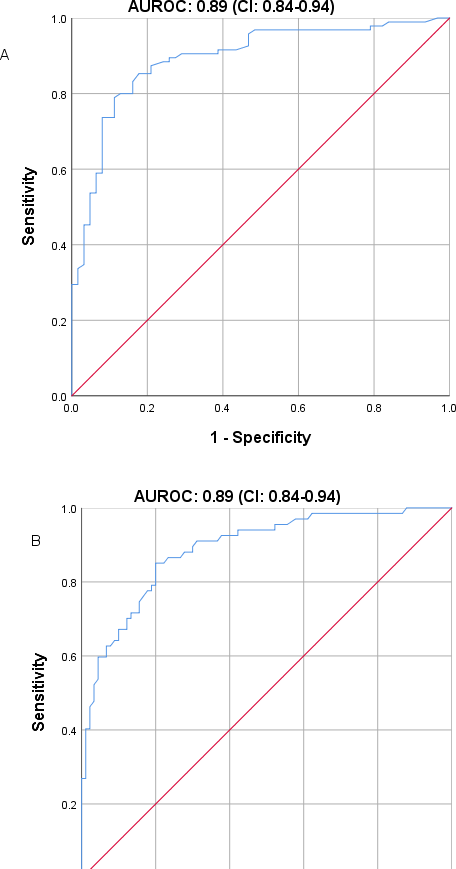

Supplement: Supplementary file 3 — Supplementary Information 3. [file 41598_2021_98776_MOESM3_ESM.png]

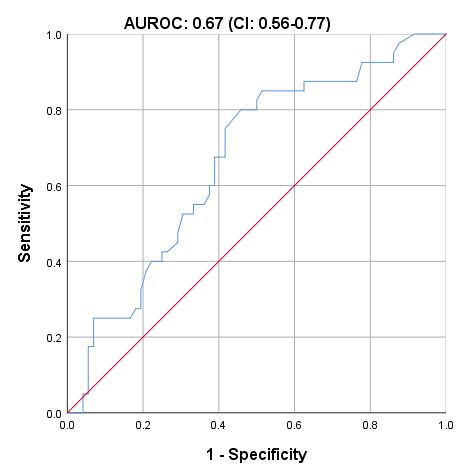

Supplement: Supplementary file 4 — Supplementary Information 4. [file 41598_2021_98776_MOESM4_ESM.png]

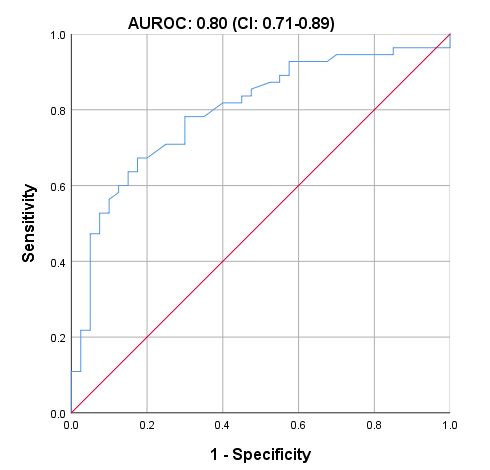

Supplement: Supplementary file 5 — Supplementary Information 5. [file 41598_2021_98776_MOESM5_ESM.png]
